# Book Reviews

**Published:** 1833-09

**Authors:** 


### CRITICAL ANALYSES.

Quæ laudanda forent, et quæ culpanda, vicissim,

Illa prius, cretâ; mox hæc, carbone, *notamus.*—PERSIUS.

*An Inquiry into the Causes of Respiration; of the Motion of the Blood, Animal Heat, Absorption, and Muscular Motion: with Practical Inferences.* By JAMES CARSON, M.D., Liverpool. *Second Edition.*—8vo. pp. 447. London: Longman and Co.

PHYSIOLOGY could scarcely afford a more interesting or important series of investigations than those which Dr. CARSON has proposed to discuss in the several essays included in this volume. The title-page is full of promise, and from those which follow, though not all that we could wish, much information may be gathered.

Dr. Carson has long been well known to the public as an industrious and original experimenter, and accounts of his researches have from time to time appeared in various periodical works; they have been subjected to severe examination, and the justice of most of his conclusions subsequently admitted. The present work is not merely a reprint of the former treatise, nor of the successive monographs alone, but the whole matter has been re-arranged, various parts re-written, several added, and it may now be esteemed the matured exposition of the author's ideas on the very important subjects on which it treats, viz. *the causes of respiration, the motion of the blood, animal heat, absorption,* and *muscular motion;* neither are the *practical inferences* added without their value.

A brief account of the structure of the thorax introduces the first inquiry, and, "as the causes to which the ascent of the diaphragm is attributed in the following pages are altogether different from those to which that movement has hitherto been ascribed by physiologists; as those causes are supposed to discharge an important part, not only in the process of breathing, but also in the circulation of the blood, and in other functions of the living machine," the author, in the first place, endeavours to shew the utter improbability of the commonly accepted doctrine. He says,

"When the diaphragm is contracted, as is acknowledged to be the case in inspiration, the oblique and transverse muscles of the abdomen are supposed to be in a state of relaxation; and, by permitting the abdominal viscera to protrude, to allow an easy descent to the diaphragm. When however the diaphragm becomes relaxed, either with or without the consent of the will, the abdominal muscles, either in consequence of sympathy, association, or of the stimulus of volition, contract in their turn, and, by pressing the abdominal viscera against the lower or concave surface of the diaphragm, cause it to assume a higher elevation in the cavity of the chest.

"The abdominal muscles and diaphragm are considered in this explanation to act as antagonists; and it is by the alternations in the contraction and relaxations of these muscles and the diaphragm that the function of respiration is supposed to be performed.

"That the actions of the abdominal muscles should perpetually and invariably alternate with those of the diaphragm, through sympathy, habit, or association, and generally without the intervention of the will, is a doctrine which appears so incomprehensible, that it will not slightly obtain the assent of the inquisitive mind. Great support has been supposed to be derived to the hypothesis of such a connexion subsisting between the muscles in every respect distinct, from the alternation in the movements of the ventricles and auricles of the heart. But the aid which this doctrine is supposed to receive from the analogy of the heart is in this case fallacious; for, by referring to the account which will be given in the succeeding part of this work, of the action of this organ, it will be found that the alternations to which allusion has been made are not produced through sympathy, habit, association, concatenation, or any of that tribe of causes, but that they are the result of a mechanical necessity.

"But this doctrine, so improbable in its own nature, receives little or no support from observation. The muscles of the abdomen are not generally during expiration in that tense state, which their contraction to the extent supposed would render necessary. Nor do the abdominal viscera appear to sustain greater pressure during expiration than at any other period.

"In cases of great inanition and collapse of the bowels, the contraction of the abdominal muscles, by bringing those muscles nearer to the direction of straight lines, passing between their attachments, the brim of the pelvis and the lower ribs, would not push the viscera of the abdomen against the diaphragm; but, by enlarging the cavity of the belly to a capacity beyond that which these viscera required, would draw them from the lower surface of the diaphragm. When the bowels are found protruded in consequence of extensive wounds in the belly, respiration proceeds for some time without more interruption than may be supposed to be derived from pain. But the diaphragm in these cases could not be elevated by the pressure of the abdominal muscles, which, during their contraction, would more easily displace the bowels to a greater extent than elevate the diaphragm. In cases of impaling, and in the numerous experiments which have been made upon the lower animals, it is found that respiration may be performed for a considerable time, after the abdominal muscles have been divided, or altogether removed. After death even, when, in dissection, the abdominal muscles have been divided, and the viscera have been taken from the cavity of the belly, the diaphragm is observed to preserve a great convexity, attended with a drum-like tenseness, towards the chest.

"If we consider how improbable it is, that a perpetual and invariable alternation in the actions of the abdominal muscles and diaphragm should proceed from the supposed causes; that a doctrine so incredible in itself receives little support from observation; and that the effect, which is supposed in all cases to arise from this cause alone, is certainly produced in circumstances in which that cause cannot operate; we may conclude with the utmost safety, that the contraction of the abdominal muscles is not necessary to the elevation of the diaphragm, and that, in general, it has no share in that effect." (P. 4.)

The elasticity or resilience of the lungs, is the cause to which Dr. Carson attributes the ascent of the diaphragm.

"The substance of the lungs is powerfully elastic. This is admitted by all anatomists. If a piece of the substance of the lungs be taken while its structure is still perfect, and stretched into double or treble its ordinary length, it will, upon the removal of the distending force, resiliate briskly into its former dimensions. This property was confidently to have been presumed from the cartilaginous and ligamentous structure of the bronchial vessels.

"From the moment that the first inspiration is drawn by the infant, to the close of life, and after it, so long as the structure of the frame is uninjured, the lungs are swelled into a forced or unnatural state of expansion; that is, they are distended into dimensions far more ample than those which they would assume, if all force or restraint was withdrawn from them. The power by which they are kept in a constant state of forced dilatation, is unquestionably, what has been stated, the weight of the atmosphere." (P. 13.)

A variety of experiments are detailed, by which it has been endeavoured to ascertain the power of elasticity, or the degree of resiliency, that the lungs possess. We shall content ourselves with quoting one as an illustration, referring to the volume for the more full account.

"On the same day another bullock, with a less capacious chest, was made the subject of experiment with the same apparatus. Water was poured into the apparatus, as in the preceding instances, till it stood sixteen inches above the level of the water in the globe. An opening was then made into the abdomen. A fold of the fleshy part of the diaphragm was then drawn down on each side, and care being taken that no part of the lung was included in the fold, it was cut into. The sound of a current of air pressing through the openings into the chest was distinctly heard, and the water in the upright tube rose instantly to the height of eighteen inches above the level of the water in the globe. The diaphragm, before the openings, was still tense, and slightly concave towards the belly; but after that, it became lax, wrinkled and flat. Some additional water was poured into the apparatus; but we were prevented in this case, as in the preceding, from ascertaining the amount of the force requisite to distend the lungs to the dimensions which they usually occupy in the living animal, by the deficient capacity of the glass globe.

"By these experiments I think it is clearly ascertained, that the spring of air compressed by a column of water of a foot and a half high, is not equal to the resilience of the lungs of an ox, at the usual stage of their dilatation." (P. 25.)

Similar experiments on dogs, cats, and calves, were attended with similar results, and corroborated the previously expressed opinion; and the author concludes this subject by observing,

"In the various examinations which my opinions have undergone, the existence of this power has been admitted, and the claim to priority in the detection and application of it freely conceded to me; but it has been contended, that the amount of it, in some instances, is inconsiderable, and consequently, that the effects ascribed to it have been greatly overrated. By these experiments, the power has been proved to be greater than my anticipations even made it, and fully adequate to all the important offices which I have ventured to assign to it. From a defect in the apparatus, the extent of the power in question could not be ascertained in the lungs of oxen and animals of their size; but it was proved to exceed considerably the force necessary to support a column of water of a foot and a half in height above its level. In calves, sheep, and in large dogs, the resiliency of the lungs was found to be balanced by a column of water, varying in height from one foot to a foot and a half; and in rabbits and cats, by a column of water varying in height from six to ten inches." (P. 30.)

Next follow the inferences drawn from these experiments, which include the *new theory* of the causes of the ascent of the diaphragm, and, as a consequence, the general causes of respiration.

"It has been stated, that, during the life of the animal, and after death, until an opening shall have been made into the cavity of the chest, the diaphragm assumes the form of a cone, and that the causes of this phenomenon would be afterwards pointed out. The brief explanation which is now to be given of this appearance will afford, at the same time, a perspicuous view of some of the important purposes to which, in my opinion, nature has turned the elasticity of the lungs.

"While the chest is in a sound state, a balance of atmospherical pressure ponderates against the external surface of its walls; or these are pressed inwards more than they are pressed outwards, by a given weight. The shell of the chest possesses sufficient stability to resist this pressure without changing in any considerable degree its form and capacity at all parts, except at the base, or diaphragm; which being muscular, pliant, and of a more extensive area than that of the transverse section of the chest, is, in consequence of the greater weight sustained by its outward or inferior surface, necessarily pressed, or, in popular language, sucked upwards in the form of a cone. The extent of this cone will be necessarily regulated by the extent of the area of the diaphragm, compared with that of the area of the transverse section of the chest. But the contraction of the muscular fibres of the diaphragm diminishes its area, and reduces it to a nearer equality with the area of the transverse section of the chest, and thus diminishes the magnitude of the diaphragmatic cone, and in an inverse proportion enlarges the boundaries of the chest. But the diaphragm, at the succeeding relaxation of its fibres, is restored to its former dimensions, becomes capable of being swelled into a larger cone, and, by this encroachment, reduces the boundaries of the chest to their former limits.

"Two powers are therefore concerned in regulating the movements, and in varying the dimensions and form of the diaphragm, the elasticity of the lungs, and the contractile power of the muscular fibres of the diaphragm. Of these powers, the one is permanent and equable, the other variable and exerted at intervals. The contractile power of the diaphragm, when fully exerted, is evidently much stronger than its antagonist, the resilience of the lungs; but the latter, not being subject to exhaustion, takes advantage of the necessary relaxations of the former, and rebounding, like the stone of Sysiphus, recovers its lost ground, and renews the toil of its more powerful opponent.

"Breathing is in a great measure the effect of this interminable contest between the elasticity of the lungs and the irritability of the diaphragm.

"The cause of the successive contractions of the diaphragm, in those cases at least in which the will is not concerned, seems to admit of the following explanation. A permanent and invariable load is sustained by its lower surface. By this load the relaxed muscular fibres become stretched to a degree which at length becomes painful and stimulating. To relieve itself from this irksome burden, the diaphragm is roused to contraction; but this contractile power, agreeably to the laws of muscularity, is soon exhausted, and, falling into a quiescent state, allows the painful and stimulating distension of the relaxed fibres of the diaphragm to be again renewed. From the irksomeness of this condition it relieves itself by a fresh contraction. Thus, by the alternated superiority of two powers, on the balancing of which life itself depends, the chest is successively enlarged and diminished, and air alternately expelled and inhaled.

"Such is the process of respiration, so far as it depends on the movement of the diaphragm; but, according to a provision very common in the animal machine, this important office is not intrusted to one class of organs alone, but has a double class assigned to it. The objects obtained by this provision are, in the first place, that by the united action of both classes, the function may, in the most healthy state, be more efficiently performed; and, in the second place, in case of the destruction or deficiency of one class, it may be still continued wholly or chiefly by the other. The second class of organs, as they may be called, discharge their office by alternately increasing and diminishing the width of the chest. This class comprises the intercostal muscles, or short muscles, which pass obliquely between the ribs, and have one insertion in the lower edge of one rib and the other in the upper edge of that next below it, and several of the muscles of the chest; and the antagonists of these agents, the cartilages, which unite the ribs to the breastbone or to each other, and the lungs." (P. 30.)

It will be seen, from the foregoing extract, that the subsidiary uses of other parts are not overlooked by Dr. Carson, although the resilience of the lungs, and the contraction of the diaphragm, are esteemed the principal causes; and, in some arrangements of the mechanism, the secondary organs chiefly perform the duty.

"It has been stated that a double set of organs belong to respiration. It happens very frequently in this country, perhaps more than in any other, that the lungs become diseased to such a degree as to be deprived of their elasticity. This may happen to one lung, and not to the other, and to one portion of a lung, while the rest remains sound. When both lungs are deprived of their elasticity, the diaphragm is deprived of the power by which, in ordinary circumstances, it is elevated, and from that time ceases to have any share in the process of respiration. In this case, which occurs when the lungs become completely tuberculous and schirrous, respiration is performed solely by the intercostal and other muscles of the chest, and their only remaining antagonist, the elasticity of the cartilages of the ribs. It occurs occasionally, particularly in old age, that the cartilages of the ribs lose their elasticity, by being converted into inelastic bone. In that case, breathing is performed solely by the play of the diaphragm. In some animals, one set of organs are used almost solely, or more extensively than the other. In animals of the chase, respiration is chiefly performed by the movement of the diaphragm. This is evident from the violent action of the flanks. The lungs of these animals are large, powerfully elastic, and little subject to disease. In the human species, again, the second set of organs are much employed, which is evident from the fascinating heaving of the female chest.

"In consequence of the descent of the diaphragm, and simultaneous elevation of the ribs, it is evident that a part of the pleura pulmonalis, that was opposite to, and in contact with, a part of the pleura costalis in the stage of expiration, will not be opposite to, and in contact with, the same part in the highest stage of inspiration, but will be opposite to a part considerably lower in the chest; and this difference between the parts of apposition in these stages will increase with the descent of the chest. Hence arises a sliding motion of the surface of one pleura on the surface of the other pleura; a motion which will be more extensive the nearer an approach is made to the diaphragm. Hence it happens that, in the healthy state of the organs of respiration, particularly in the healthy state of the lungs, no adhesions can take place between the pleura.

"The lungs themselves, like the organs of respiration, of which they constitute an important part, are double, so that, if one lung be rendered inefficient, by disease or otherwise, respiration may still be performed by the aid of the other lung only. Hence it happens that one lung may be completely collapsed without much apparent inconvenience to the animal. I found, by some experiments which I made on animals some years ago, that the air may be admitted freely upon the lung of one side, without the destruction of life, and indeed with perfect safety. This proves that the mediastinum, or membrane placed between the two lungs, and dividing the chest vertically, is sufficiently tense and strong to be a wall to either of the lungs, too powerful to be overcome by the resilient effort of that lung, and to secure to it a space sufficiently ample to enable it to perform the purposes of respiration. A very striking and satisfactory proof of this, and indeed as conclusive a one as has yet been adduced, of the extent of the power generated by the elasticity of the lungs in a state of health, is supplied by the manner in which the Jews kill the animals intended for their food. It is well known that this people will not eat the flesh of any animal that has the blood in it, or that has had any disease or malformation of its organs. A mode of killing animals different from that in use with Christians, and a peculiar examination for ascertaining the health and perfection of the organs of the animal, are practised by them. To separate the blood as completely as possible, the operation of slaughtering commences with bleeding, which is done by dividing all the vessels of the neck, including the windpipe. In this situation the animal bleeds to death; a tedious, and apparently a very cruel process. The operator, who is generally the priest, or some person appointed to this sacred duty, then proceeds to the examination of the viscera. The abdominal cavity is first freely opened, and examined. He next proceeds to examine the state of the thoracic organs: for this purpose he makes a large incision through the diaphragm on the right side. On the first occasion of this kind at which I was present, an ox happened to be the victim. Upon the incision being made through the diaphragm, a loud sound, like that produced when a large drum is struck, was heard, for which I was not prepared, and which startled me like the unexpected firing of a pistol. This sound was occasioned by the rapidity with which the air rushed through the orifice to occupy the space left by the collapse of the lung. The sound being in this instance rather louder than usual, was regarded by the operator as a proof of the soundness of the thoracic viscera. He next perforated the diaphragm on the left side: a sound, though not so loud as that following the first incision, was distinctly heard at some distance. The extent of the sound in the first instance proved the force and rapidity with which the lung collapsed; the sound in the second instance, of diminished extent, proved that the mediastinum had yielded to a certain degree, but that it had preserved its position so far as to check the force derived from the elasticity of the lung upon its left side, after that lung had been slightly collapsed, and to preserve that lung in a condition fitted to carry on the process of respiration.

"It is chiefly to the motions of the diaphragm that the respiration is to be ascribed; though, as has been stated, in some animals, and especially in man, a considerable share of this office is to be attributed to the intercostal muscles. Those animals which are remarkable for swiftness and perseverance in the race scarcely employ the intercostal muscles, but use the diaphragm almost solely in breathing.

"The action of the abdominal muscles, though not necessary, may occasionally be useful in respiration. When any substance lodges in the bronchi which is too tenacious to be separated, or too ponderous to be elevated by the powers commonly employed in respiration, the contraction of the abdominal muscles will increase the pressure against the lower superficies of the diaphragm, and augment the velocity and the force with which the air passes through the bronchi and the windpipe.

"There may be also many diseases of the chest in which the action of the abdominal muscles may be serviceable in respiration.

"In no circumstances almost, during health, can it be supposed that the aid of the abdominal muscles is required to elevate the diaphragm, as the elasticity of the lungs is of itself sufficient for that purpose; and, unless it were counteracted by the residue of contractile energy subsisting in the muscular fibres of the diaphragm even during their relaxation, the cause might often be found too potent. In the act of sneezing, when the diaphragm, after a full contraction, is suddenly seized with a kind of paralysis, it is probable that the unresisting fibres are protected from the injury which they would be in danger of incurring, in consequence of the violence of the jerk to be sustained in their relaxed state from the inequality of pressure, by the synchronous and almost involuntary contraction of the muscles of the larynx, fauces, and mouth. By this contraction the free exit of the air is resisted; the air in the chest, confined and compressed, checks the collapse of the lungs, and of consequence the ascent of the diaphragm.

"This defence, however, is not always effectual. Sneezing has been known to be suddenly fatal, in consequence, as has been supposed, of the rupture of a blood-vessel in the chest, but more probably of the rupture of the diaphragm itself." (P. 38.)

These physiological researches suggested the idea that a surgical operation might be serviceable in certain thoracic diseases, and especially in pulmonary consumption; and, although no very beneficial effects followed the two operations that were performed, still, as being founded on rational principles, however bold and energetic the practice may appear, we shall quote the details entire, and recommend them to serious consideration.

"As satisfactory proof was supplied by repeated experiment, that an animal could live by using one lung only in respiration, it was imagined that the knowledge of this might be applied to the cure or relief of some diseases, particularly of consumption. It is generally believed that the ulcers, from which matter is discharged in consumptive cases, are prevented from healing, as well as the same kind of ulcers would do in other parts of the body, by the agitation to which they are exposed in respiration.

"In addition to that cause, another powerful one was supposed to be derived from the structure of the lungs, and from their position in the living body. In that state, the elastic substance of the lungs being always on the stretch, would be distended beyond its natural limits; if therefore the fibres of which the lungs were composed were divided, they would resiliate from each other, and the wounded surfaces could not again be brought into contact.

"It appeared to be practicable to remove those two causes of the impediment to the cure of wounds, by reducing the diseased lung to a state of collapse. This would evidently be done by admitting the air into the cavity of the chest, by an opening through the side, and preserving the passage to it free for a sufficient time. The lung would by those means be reduced to a state of quiet collapse, and the elastic fibres separated by the wound would be brought together. The lips of the divided surface would be brought into contact. The disease, if in one lung only, as frequently happens, would be cured by this process alone.

"It was found, that if a lung which had been reduced to, and kept in, a state of collapse for some time, and if the opening, through which the air had been admitted for reducing it to that state, were allowed to heal, the lung would, in consequence of the absorption of the air contained between the two pleuræ, be again dilated to a contiguity with the internal surface of the chest; and that, if the other lung were diseased, it might then be with safety reduced to a state of collapse; depending upon the lung now healed, for the performance of respiration; and treated as the former had been. A paper containing those speculations was read in the Literary and Philosophical Society of Liverpool, where it produced a considerable sensation, and raised hopes, which, alas! have not been realised, both in several members of the medical profession, and other members of the society. An opportunity occurred some time after, without any solicitation on my part, of putting the matter to the test of experiment.

"James Sloane, Esq., an eminent merchant of Liverpool, the last of five brothers, the other four having died of consumption a few years before, had returned from the West Indies, to which he had gone for the purpose of trying what a change of climate might do in his case, in the last stage of consumption, which he knew to be incurable from any known remedies. Soon after his return, he heard of the paper to which I have alluded, and soon became determined of having the operation, which I had suggested as a possible means of giving relief, performed in his own case. It was done on the 26th of Sept. 1822, by Mr. Bickersteth, an eminent surgeon of this place, in presence of the late Dr. M'Cartney and myself. An incision calculated to admit air freely into the chest, was made between the sixth and seventh rib. As the sound usually heard upon an opening being made in the chest, and produced no doubt by the rapid passage of the air through the opening, was not perceived in this case, it was suspected that the lung did not collapse, and that an adhesion had prevented the entrance of the air. It was not deemed advisable to make a further examination for that purpose at that time; a tent was inserted into the wound to prevent an adhesion of the sides, by the first intention. At our visit, next day, when the wound was examined, our suspicion of adhesion between the lungs and the chest at the place, was confirmed at the first aspect; the discharge from the wound had not found access into the cavity of the chest, which it ought to have done in the position of the body, but was oozing out of the wound; an examination by the finger ascertained an adhesion of the lungs to the ribs, both above and below it; a probe found admission to a greater length across in both directions, but at length met with an obstacle. Mr. Sloane suffered no inconvenience from the operation, but thought his chest somewhat relieved. This might happen from the adhesion being rendered by it less tense. The orifice was kept open, and he suffered no inconvenience from it till the day of his death, which happened about a month after the operation, and which had been deferred fully as far as it was suspected it could be, at the time he arrived from abroad. Examination after death, shewed adhesion to have taken place between the pleuræ completely at the diaphragm, along both sides of the mediastinum, and along the chest, every where except at the spaces between the ribs, where the fingers might be admitted as into the fingers of a glove. The lungs filled the chest, and shewed no tendency to collapse. Two large abscesses were found, one in each lung. The substance of the lungs was tuberculous and hard throughout. Adhesion, so universal, did not in this case appear to arise from any previous inflammation, to which these adhesions are generally attributed; for, though I attended him regularly in every complaint to which he was subject for many years, I never had reason to suspect any inflammation of the chest; but, in my opinion, from the tuberculous affection of the lungs, by which they were deprived of all elasticity, and of consequence of that sliding motion above described, which occurs in health, between the pleuræ, and which I conceive to be the chief cause of the absence of adhesion between these membranes. The diaphragm was deprived of its antagonist, the elasticity of the lungs. The customary movement of that organ must long have ceased, and in its quiescent state it had readily grown to the pleura pulmonalis. It was observed long ago, by that great ornament of the medical profession, Boerhaave, of Holland, to be in phthisical affections a bad symptom, if the abdomen did not protrude in inspiration, or in other words, if the diaphragm did not descend. The cause of the unfavorableness of this symptom, of which Boerhaave could have no just knowledge, can now be satisfactorily explained.

"After the death of Mr. Sloane, the attention of myself, and indeed of the medical profession of Liverpool, was directed more earnestly to the condition of the lungs in consumptive cases; and it was found universally, when patients had died of tuberculous phthisis, that the lung affected did not collapse in its whole extent, and that adhesions had uniformly taken place between the pleuræ. It often happens that only one lung is diseased; in that case, no adhesion will be found to have taken place in the side occupied by the sound lung. If the lungs were tuberculous, but not altogether solid; if some elasticity still remained, the adhesions would be confined to the upper part of the chest, where the sliding motion is at all times slightest, and leave the lower part of the sides and the diaphragm free. Adhesions in the chest, I would conclude then, do not at all times arise from inflammation, but perhaps most frequently from a disease in the fabric of the lungs. It is very evident that the operation which was performed on Mr. Sloane could have no good effect in the case of tuberculous consumption. Whether there are any other kinds of phthisis in which it might be serviceable, will afterwards be examined. Let us inquire, now, whether any thing further could be done in such a disease as that of Mr. Sloane; I am of opinion that something more might be done, and that a dependent opening might be made through the ribs, for the discharge of the matter. If it is supposed the operation had not stopped at the division of the pleura costalis, but had made an opening into the abscess through the substance of the lung itself, no injury could evidently arise to respiration by such an operation. In this state of the lung, it signified nothing whether the air passed through the windpipe or through holes in the sides. The lung could not be collapsed. I anxiously waited for an opportunity for making this operation; I resolved not to make the proposal, but would encourage it in case it came from the patient himself, or some of his friends. An opportunity of this kind occurred in an intelligent merchant of this town, of the name of Johnstone. He was well acquainted with the nature of the operation that had been performed on Mr. Sloane. I explained to him what appeared to me requisite to give it any chance of success. Finding himself on the brink of the grave, he proposed that the operation should be performed to the extent that I wished. Mr. Bickersteth, who, it is only fair to state, had shewn as great zeal in this scheme as if he had been the original projector, was selected as the operator. The patient's own surgeon, Mr. Samuel M'Culloch, was, at the request of the patient himself, present. It was agreed that the operation should be proceeded in after the same manner that it had been done in Mr. Sloane's case; and, if matters were found in the same condition as they were in that case, to cut freely into the substance of the lungs, taking care that the large blood-vessels were avoided. When the first part of the operation was performed, matters appeared to be exactly as they were in Sloane's case. This was explained to the patient, but he declined having any thing more done at that time. At a subsequent visit of Mr. Bickersteth, at which I was not present, the patient consented that the operation might then be completed, which Mr. Bickersteth, taking advantage of the present disposition of the patient, instantly complied with. An incision was made into the lung. A pretty profuse hemorrhage took place, which the operator had considerable difficulty in stopping. No abscess had been reached, no purulent matter was discharged, the disease pursued its course, and in a few days terminated with the death of the patient.

"The appearances on dissection were exactly the same with those which had been exhibited in Mr. Sloane's case. The lungs adhered everywhere to the substances that were in contact with them, except at the interstices between the ribs. The incision which was made on the right side had nearly reached a large sac full of purulent matter. It may be asked, what benefit could have arisen to the patient if this sac had been opened, and an outlet afforded to its contents through the side? I answer, great. In the first place, a dependent opening would have been afforded to the matter in the sac, from which it would have been discharged as soon as formed, instead of accumulating, lodging, and constantly filling the sac. It is well known how advantageous such openings are in parts externally situate. The matter, so far as that sac was concerned, would have been discharged without coughing: the painful and exhausting nature of the process required for discharging it in this manner is well known. The sores in that lung would have been placed in a situation as favourable for healing as the same sores could have been in any other part of the body. Nor would it have been necessary to have confined the operation to one side. The breathing on the side on which the operation had been made would have been rendered less imperfect, insomuch as it would have been relieved from a load of morbid and acrid matter.

"As I have before remarked, since the lung in this situation could not collapse, it matters not whether the air entered into the chest through the windpipe, or through the hole in the side. If coughing, and a discharge of matter by that distressing process, still continued, it was with certainty to be inferred that there was a collection of it in the other side. An outlet to the matter on this side might be easily afforded, in the same manner as had been done in the other, without increase of danger, and with the same relief to the respiration. If no matter were discharged by cough, it was to be inferred that the other lung was sound, and no further proceeding necessary.

"It will readily occur to any one, and appear as an objection to the operations which have been stated, that the matter of a diseased lung is not contained in one sac, but in a multitude of tubercles, without having communication with each other. It may be remarked, that the matter from each of these sacs finds its way, sooner or later, into some of the ramifications of the windpipe; and, as those ramifications have a communication with each other by their common stem, a communication between this stem, or indeed any of its branches, and the outlet in the side, would secure the discharge of matter as certainly through this outlet as through the windpipe; and, a communication being once established, it might be rendered more free, as the discharge was encouraged to take that course.

"In cases of empyema on one side of the chest, a dependent opening for the discharge of the matter contained between the pleuræ would be of indisputable benefit; and, as it might be ascertained by symptoms rendered more plain by the use of the stethoscope, on what side the matter was contained, and as empyema arises frequently in constitutions in which the lungs are otherwise little diseased, complete cures might be often expected.

I remember the case of a lady who fell a victim to this disease, who, I think, might with certainty have been cured by an opening made into the chest. She discharged periodically, I think thrice a day, about a gill of matter. This matter was raised at once by a violent paroxysm of coughing, with great sense of suffocation. In the intervals she was tolerably well. After death the body was examined: the left sac of the pleura was filled with matter; the left lung was small and dense. It was supposed by the surgeon that the lung was wasted by the discharge of matter; but I suspected at the time that the smallness of the lung was occasioned by its complete collapse, for a long time the air being totally expelled from the bronchi. The matter found its way into the windpipe through its left branch, and when all the chest was full to the extent of its reaching this orifice, it was discharged in the manner stated. She was a lady of a very sound constitution. The disease had originally been the effect of a simple inflammation of the chest.

" No disease has been inflicted upon mankind that is more to be deplored than consumption, whether we consider the number of its victims, the length, and frequently the severity of their sufferings, the age at which they are usually selected, or the moral and intellectual qualifications with which they are commonly endowed. It crops the flower of the human race. In no way, therefore, could man be supposed to approach more nearly to the exercise of the divine functions, or to render a higher and more endearing service to the world, than by discovering a cure for this disease." (P. 51.)

We think Dr. Carson far less conclusive in his theory of *the motion of the blood,* especially of the venous circulation, than in his exposition of the mechanism of respiration; but, as our limits will not allow us to enter into a full critical analysis of his views, we rather leave this essay untouched, than treat it in a cursory manner; and this we are the more especially inclined to do, as the subject has been so often brought before the notice of our readers, and from some of the controversy upon the disputed points having been carried on in this Journal. [*See* Dr. Arnot's *paper, published in the volumes for* 1827, *et passim.*]

Much ingenuity, we allow, has been displayed by Dr. Carson in opposing the objections which have been offered to his theory; but, notwithstanding all he has said, we are among those who think " that the supposed use to which the elasticity of the lungs is converted, that of aiding the full expansion of the heart, is unnecessary, and therefore not likely to be so employed: at all events, that it cannot be considered indispensable; because the blood circulates in fishes, in birds, and in the fœtus in utero, in which lungs are either altogether wanting, or are not elastic; or, if elastic, are so situate. that their elasticity can have no avail in the way supposed." (P. 196.)

The want of synchronism between the acts of respiration and the motions of the heart; the power of rendering the respiration slower and more quick, and of suspending it for several seconds, without varying or suspending the actions of the heart, we think insuperable objections to the doctrine; and the respiration of birds seems to us an irrefragable proof of the correctness of our views. Dr. Carson himself appears conscious of its force; for he says,

"Birds, however, are not only placed in the same element with the mammalia, but are accustomed to ascend into regions in which the air is much rarefied, and, in point of specific gravity, still more remote from the blood which they carry in their veins than that which the latter breathes. The reasons by which the want of elastic lungs in fishes is accounted for, will not apply to them. An adaptation to the aërial residence which these animals have assigned to them requires a peculiarity of structure, and a different series of powers. The same functions are necessarily performed after a different plan. To enable the reader to comprehend fully the view which we have of the method by which nature has contrived to supply the office performed by elastic lungs in the mammalia, would require a more detailed explanation than can be properly given at this place. I have therefore to request the reader to suspend his judgment till the respiration, and other functions of birds, shall have been considered; assuring him, in the meantime, that it is confidently hoped that a satisfactory cause will be shewn for the deviation from the structure of the mammalia; and that, in that deviation, proofs will be adduced of some greater power being required than that derived from the simple relaxation of the heart, for securing the expansion of that organ in all animals in which a fluid circulates of greater specific gravity than that in which they are immersed." (P. 198.)

Courteous as we wish to be, we cannot, if pressed, "suspend our judgment;" and on the present evidence it would, we confess, be given against our author. When, however, he brings forward the "*satisfactory cause*" which he "*confidently hopes*" may be shewn, we shall be ready to review the verdict, to grant him a new trial, and, if "*satisfactory cause*" be shewn, to reverse our sentence.

*Clinical Lectures on the Contagious Typhus, epidemic in Glasgow and the Vicinity, during the Years* 1831 *and* 1832. By Richard Millar, M.D., Senior Physician to the Royal Infirmary, and Regius Professor of Materia Medica in the University of Glasgow.—8vo. pp. 184. Brash and Co., Glasgow; Black, Edinburgh; Longman and Co., London.

(Concluded.)

The observations on prognoses are concluded with some remarks on those derived from sleep and age. Of the first the author says,

"That this important function of health should be always more, or less, impaired, by the numberless pains, or uneasy feelings, that harass the sick in typhus, was necessarily to be expected, but the amount of the evil is different, in different individuals, and we judge of the danger by the degree. If rest be little interrupted, it is needless to say that the sign is propitious, and if entirely wanting, no less the contrary, the latter circumstance invariably adding to the exhausting power of fever, and, of course, robbing the patient of that reserve of strength which might, otherwise, enable him to bear up under the pressure of so protracted a disease. Besides, almost all the symptoms are sufficient, individually, to banish sleep, and when sleep returns, it may be hailed as a signal that these are surmounted, or, at least, have abated of their violence. On all these accounts, the state of sleep, as forming an important element in prognosis, is always an object of particular scrutiny in the chambers of the sick.

Here, however, I must inform you, that on this point you cannot always trust to the report of the sick themselves. Say that you inquired of a patient how he rested during the preceding night, he will not unfrequently answer, that he never shut an eye, though you are informed, and informed truly, by the nurse, that he passed the whole time in a state of quiet and comfortable repose. There is here no intention on the part of the sick person to deceive you, he is, in fact, deceived himself. He labours under the influence of a particular species of delirium, or coma, not unusual in the disease, which has been not unaptly termed *coma vigil*, by authors. This singular symptom may perhaps receive some elucidation from the remarks that follow. Without entering here into any intricate discussion concerning that curious phenomenon of living bodies we are accustomed to designate by the name of sleep, and of which a rational theory is alike wanting to metaphysics, and to medical philosophy, it will be sufficient to remark, that one of the principal means by which we distinguish our waking, from our sleeping hours, is, by the vivacity, or strength, of the impressions of sense, as they flow directly from their immediate organs. contrasted with the faintness, or indistinctness, of the same impressions as afterwards converted into their corresponding ideas, and reflected back by the imagination, and memory, two faculties, be it remarked, that lose but little of their activity, and vigour, during the time of our nocturnal repose. The same criterion, too, during health, prevents us from confounding dreams, even the most vivacious, with actual occurrences. But this power of discrimination is occasionally lost in disease. In typhus, memory, and imagination, are no less disordered than other sensorial functions, and their particular perversion here seems to consist in this, that the ideas they present to the mind are so preternaturally, or morbidly, vivid, that they pass with the patient for the genuine and original impressions of his external senses; and hence, under this delusion, he is induced to deny that he slept at all, during the night, but, on the contrary, to maintain that he passed every hour broad awake. His whole train of thought was one continued dream, but a dream so impressive, that it was readily mistaken for reality. Such sleep, however, imperfect as it may be, is better than none. It at least secures rest to the voluntary muscles, and exempts from the tædium and misery of entire wakefulness. As amendment proceeds, this excited state of the imagination, and memory, gradually wears away, and along with other functions, they recover their former soundness, so that this impediment to the natural state of sleep no longer continues. A return to the former enjoyment of rest, indeed, may be viewed as, at once, an effect, and cause, of convalescence. As the first, it shows the declining state of the disease; as the second, it exerts a kindly influence over the remaining symptoms, more especially renovating, and restoring, the lost, or languishing, powers of digestion." (P. 25.)

On the second he observes:

"It is a curious and certain, but hitherto unexplained fact, that at the beginning of life, typhus commits much fewer ravages than afterwards: indeed, at this era of existence, it seems almost entirely harmless. At what particular year, however, this exemption from the danger of fever is enjoyed in the greatest perfection, when it commences, or when it terminates, I am unable to inform you; yet I think it may be safely enough affirmed, that previous to seventeen, a patient, comparatively speaking, runs very little risk from this malady. Of this assertion, some no less satisfactory than striking proof, may be adduced. Thus, in the Cork Fever Hospital, nine hundred and fifty-five patients under seventeen years of age, were treated by Dr. Barry, and only six died, or 1 in 159 and a fraction. I confess, when I first read this statement, it appeared to me so extraordinary, that I was led to suspect one of two things, either that the cases had not been all instances of genuine fever, or that there was some error in the printed numbers. Now, however, I believe in the correctness of the account implicitly, and you have all witnessed full corroboration of it in our own wards. Here you have seen treated two hundred and sixty patients, under seventeen, one hundred and ninety-six males, sixty-four females, and the deaths were only six; four among the former, two among the latter. But of the patients thus lost, not above two or three at most were cases of genuine unmixed fever, so as to be properly counted in the catalogue of casualties." (P. 27.)

Whether the Glasgow contagious typhus differed essentially from the fevers known by the same denomination in other places, we shall not pretend to determine. From the symptoms detailed, we should presume that it did not; and, from our author's caution in ushering in his account of its treatment, we were led to surmise that he thought similar treatment would not be approved for similar diseases in other places; that some parts of it will be discommended by many can be easily foreseen.

"We come now to the most important part of our subject,—the cure, or, more correctly speaking, the treatment of typhus.

"Here I think it necessary to premise, that the directions I am, at present, to lay before you, relate solely and exclusively to our low contagious typhus; how far they may be applicable to other forms of fever described by authors, I offer no opinion.

"The treatment of low typhus naturally divides itself into two branches, one, comprehending the measures to be followed, at the commencement of the malady, the other, those that may be called for after it has been fairly formed.

"Of the first set of measures the purpose is of the highest importance, no less than to strangle, or extinguish, the disease at its outset, and, of course, avert all future danger. Two plans chiefly have been resorted to with this view, one, the early administration of an emetic, and diaphoretic, the other, the shock of the cold affusion. With respect to the first, the process I have found most advantageous is the following. About eight o'clock in the evening, I order the emetic, generally ipecacuanha, after the operation of which, the patient is enjoined to bathe the feet and legs in warm water, during at least a quarter of an hour, or twenty minutes, and upon retiring to bed, he is to swallow a large dose of Dover's powder. Sweating soon commences, and it is to be supported and encouraged by tepid diluents. This method, when administered early enough, I have found singularly successful. My chief experience of it has been among the nurses of our Infirmary, when they happened to catch the contagion. With them, I have seen it, repeatedly, dissipate every symptom of fever.* Upon visiting them next morning, I found,

* "The young gentlemen who serve as clerks in our Fever Hospital rarely, if ever, escape the disease, and no wonder, considering the duty they have to perform. This duty consists, first, in taking a history of each case as it enters the house; an employment which causes them to linger long in near contact with each patient, and many such histories must often be taken the same day; then they must regularly visit all the wards thrice daily, besides frequent additional calls, both by day and night, so that they live almost constantly in an atmosphere of contagion. Two of them, who acted successively in this capacity for me, Messrs. Paxton and Howie, and to whose zeal in acquiring knowledge of their that after profuse sweating during the night, the pulse had come down, that the headach, with the pains of the back and limbs, had vanished, that the tongue had regained its moisture, and that nothing remained but a little debility; in short, that all those threatening signs had disappeared, that, if left alone, would have soon matured themselves into a regular and genuine typhus. It is almost needless to say, that this happy result is most looked for only when we have the fever to deal with at its very commencement." (P. 32 )

The account given by Mr. Howie is very similar to that of Mr. Paxton, and the treatment there described proved equally successful.

"Another plan resorted to for eradicating typhus fever in its first stages, is by the shock of the cold affusion. This method, however, is more circumscribed in its use than the other, since it is available only when the skin is at once preternaturally hot, and free of moisture, a conjunction not often observable in our low typhus, even at the beginning. With these requisites, however, it is a most powerful remedy, and that it has at different times extinguished fever, is a fact established by the testimony of the most trust-worthy practitioners. Of this plan I have myself little or no experience. I tried it twice only, but both times without success. Like our other expedients for shortening fever, it is too often delayed till the time for using it has elapsed, or till the disease is so firmly fixed as to defy its influence.

"It thus appears that there exists in fever what may be called a preventive stage, a period during which it remains assailable by medicine, may be effectually checked in its career, or may be utterly profession, humanity to the sick, and general good conduct, I have great pleasure in bearing this testimony, put the prophylactic plan above recommended to the test of experience in their own persons, and fortunately experienced its full benefit. Both of them warded off, by means of it, the fever that would otherwise have infallibly ensued; Mr. Paxton twice over, or on two several occasions. I subjoin the narrative of each of my young friends, in his own words:

"Dear Sir: On the 6th of August last I was seized, in the morning, with an unusual feeling of languor, and difficulty of directing my mind to my usual occupations; this was attended with considerable nausea, which continued the whole day, and I was unable to eat any thing at the usual meal times. Next morning, after having passed a very unquiet night, I awoke with severe headach, pain of back and calves of the legs, with a pulse of 108, and white tongue. I remained in bed the whole day, but at four o'clock P. M. I was seized with severe rigours, which continued for some time. A scruple of ipecacuanha taken immediately produced full vomiting. At night, after fifteen grains of Dover's powder, preceded by a hot bath, I fell into a profuse perspiration, and next morning felt considerably relieved; my pulse had fallen to about eighty, tongue still white. A calomel and jalap purge operated freely; and next day, with the exception of slight debility, all disagreeable symptoms had disappeared.

"I had a similar attack in December last, which seemed to have been arrested by the same treatment.—I am, dear sir, your obedient humble servant,

"John Paxton.

"*G. R. I. August 9th*, 1832."

extirpated. This important,but fleeting era, however, for it is only of a few days' duration, is too generally suffered to pass away without advantage, unheeded, and unimproved. The precious opportunity it affords of averting the whole future calamities of the disease, is thus completely lost. The practitioner is seldom, or ever, consulted till the after stage, when it has become too firmly entrenched to be dislodged, or when it has inseparably fastened itself upon its victim. It will then, too often, run its course in spite of every obstacle we can oppose, and this brings us to the second branch of our present subject,—those remedial means which are left us, after this change, or after the malady has thus assumed what might be properly enough termed its permanent form, or condition. Before coming to details here, I think it necessary to premise the two following axioms, together with their two corresponding corollaries.

"1. That typhus, after it has reached a certain stage, will proceed onwards in its course (a few rare instances excepted,) in spite of every obstacle medicine has yet devised to check its career; but that, at the same time, it is part of its nature to decline spontaneously, to exhaust itself, or to wear itself out by degrees.

"2. That fever is a disorder of considerable duration, and that while it endures, the whole powers of life lie prostrate before it, more especially appetite, digestion, and assimilation, so that often for ten, or even twenty days, little or no new blood can be created, or prepared, for the purposes of the circulation, while secretion, and excretion, going on continually, to greater, or less, extent, must act as a constant drain; and hence that on all these accounts, more or less languor, exhaustion or debility, must be looked for, sooner, or later, as an essential, or inseparable consequence, or concomitant, of the disease.

"From the above two axioms flow the following two corollaries, as appears to me, alike founded on common sense.

"1. That since it is beyond our power, generally speaking, to stop the current of the fever, little else will often remain for the practitioner than simply to lie by, and watch the symptoms, administering what aid he may to such as appear the more troublesome or dangerous.

"2. That, founding on the second axiom, he abstain from all inordinate depletion, more especially of blood, so as not to draw too largely on that fund of strength which ought to sustain his patient, and which seems requisite to bear him up through the usual contingencies, and exhaustion, of so protracted a disease.

"Building on the soundness of these preliminaries, gentlemen, I proceed now to more particular directions. Let us suppose, then, we have a typhus patient presented to us, and let him be an hospital patient, as over him we have the most absolute control, and let us consider what measures are to be pursued. We commence with the following. His head is first to be shaved, and his clothes being stripped off, and immediately immersed in a tub of water, so as to be completely covered, and surmounted by the fluid, he is to be thoroughly washed and cleansed in the warm bath, and, after being dried and furnished with clean body linen, he is finally to be put to bed within some large, comfortable, and well ventilated ward or chamber. We then proceed to open his bowels, a precaution never to be neglected during any period of the disease, and, as he will be generally harassed more or less with flying pains of his limbs, and with broken rest, at night, we order a dose of Dover's powder at bedtime, both to remove his uneasy muscular feelings, and to conciliate sleep. Such, gentlemen, are the initiatory measures, or common routine, I have been long accustomed to pursue. If, after all this, at our next visit, there appear no untoward symptom; if the pulse be neither very rapid, nor very feeble; if there be neither unnatural heats nor chills; if there be little headach, and no delirium; if, though the patient cannot eat, he neither purges nor vomits; if the pains of the back, and limbs, be only slight, or moderate; if sleep continue pretty regular, or be easily commanded by an opiate, I hold, that for the present, at least, little else is to be done. We may give the patient, from time to time, a little wine to relieve his languor, and it will more speedily recruit his lost strength, if it be blended with his food. Such, gentlemen, is the line of practice I would recommend to you, in all those that may be called the milder cases of typhus. Caution, and forbearance, are to be your leading maxims, here, in spite of all dashing practitioners, and all dashing books. Typhus is an enemy, you may rest assured, often to be most effectually opposed by the Fabian system of tactics, and he will be the most successful combatant, as well as best deserve the eulogy of the Roman poet, who, while he remains at his post, ready for the encounter, yet prudently abstains from all ill-timed and unnecessary attacks:

> Tu Maximus ille es,
> Unis qui nobis cunctando restituis rem.

"Dropping here, however, all figures of speech, gentlemen, I am happy to say that I have it in my power to lay before you the most complete practical proof of the truth and propriety of this precept. The treatment it recommends has not only been put to the test of experiment, but has been tried on a great scale, and, however simple, has been crowned with the most perfect success. You will find by the journals that no less than 516 patients have been treated according to this plan, that is to say, in these cases nothing seemed to be required, and accordingly nothing was done; we simply lay by, and watched the disease as it proceeded in its course. The only exceptions were, that in fifteen of the males, leeches were applied, and in five others, a blister; among the females again, neither leeches nor blisters were found necessary, while in the whole 516, setting aside these additions, the sole remedies employed were an occasional laxative, and an opiate at night. If success be the criterion of practice, the result has been most satisfactory, for out of all this multitude there was not so much as a single death."*

The precepts for the ordinary treatment of the severer forms of typhus, in which Dr. Millar does not differ from other writers, we shall pass over without extract; for, however proper in clinical lectures to students, they would look too much like truisms for our pages. But, as his estimate of opium is somewhat at variance with the common rules of practice, we shall let him state the benefits of its early exhibition.

"Among other symptoms originating from febrile disorder of the common sensory, none of the least is the privation, or disturbance, of sleep, and we are now to consider what remedies are available against this common evil of fever. For the nocturnal repose we are accustomed to enjoy, we seem chiefly indebted to that state of exhausted excitability, or torpor, which is produced, during the day, by the various and unceasing impressions of the external senses, the fatigue of our voluntary muscles, and the exhaustion produced by the ordinary affairs, or occupations of life, joined to habit, and exemption from any particular pain or uneasiness; but of these the influence is either lost, or suspended, during typhus, and hence wakefulness continues, when rest should supervene. We fly for aid to the medicines called narcotic; of all these, by far the principal is opium, a drug at once anodyne, and hypnotic, or comprising in itself the two requisites we desire,—the power of abating pain, as well as inducing that state of exhausted excitability, or torpor, which may be said to constitute, if not the essence, at least the prelude, of sleep. The utility of such a medicine cannot but be apparent. It is, indeed, a remedy of the highest importance, and, from the most ample experience, I am now so thoroughly convinced of its benefit, that, generally speaking, I would no more omit a dose of it at bedtime, than I would neglect opening the bowels of my patient by a cathartic. I say this advisedly, gentlemen, and I say it strongly, because I have observed some late writers on fever too much disposed to under-rate the virtues of this valuable article. Those who have prescribed it on a great scale, as in hospitals, will form a juster estimate of its powers, and I have often been struck

"* Those who are fond of jokes against our profession will have room here for indulging their humour. They will say, and with some plausibility, that many among those to whom we administered numerous and various medicines died, while all those who took nothing, or next to nothing, recovered. The remark is undoubtedly true, and it shows that fever will often run itself out, and terminate favourably, with little or no assistance from drugs. Still, however, we must not give up the importance and utility of our art. The previous discipline of the warm bath and shaving the head, to which all our patients are subjected, I consider of no small avail for mitigating the symptoms, and still more the removal from their own filthy and ill-aired abodes to a clean bed, and a well ventilated and comfortable apartment. Neither must we forget the maxim, that there is often more skill displayed in withholding than in prescribing a medicine."

by the testimony borne in its favour, by the poor inmates themselves of our fever wards, though in their own homely phrase, as well as pleased by the thankfulness they expressed for the relief it had afforded.*" (P. 43.)

We fully agree with Dr. Millar, that, excepting where local disease requires it, bloodletting is not to be recommended in low fevers; but we cannot accord with him, in his neglect of all other tonics and stimuli but wine and whisky.

"There will, no doubt, in every instance, be more or less disturbance of the heart and arteries, but if it do not exceed in amount what seems inseparable from the febrile state, we hold it sufficient simply to watch the patient, trusting that the disorder of the circulation may gradually subside, or wear itself out, like the other symptoms. But we are not always to look for such mild cases. In too many instances the pulse flags, becomes either immoderately quick, or alarmingly feeble, for the most part both at once, and we must then endeavour not only to restore its energy for the present, but to counteract its weakness, or tendency to sink, for the future. While on this subject, I must not forget again to warn you against the common opinion that every case of contagious typhus must always necessarily commence as a synochus, and must, on that account, be treated, at first, as an inflammatory disease. That such contingency may occasionally exist, I do not deny, but I hold it of rare occurrence; and, unless there appear some unequivocal sign of inflammation, either in the head, chest, or abdomen, whatever the pulse may be, you must carefully beware of all inordinate depletion, more especially of blood. Only have a little patience, and you will soon see the benefit of this caution. Merely watch your patient for a day or two, and you will find the hard feeling of the artery, you so much dreaded, gradually disappear, and to be succeeded by the usual typhoid beat.

"The leaning of the disease, in fact, is all on the other side, and the danger we apprehend, except in very rare instances, is not from morbid excitement, or inflammatory vigour, but from collapse, or excessive weakness. It is against this last condition accordingly that we direct our efforts. The means thus resorted to by practitioners, for counteracting the debility of fevers, have been very various, at different times. Among the old Boerhaavians who had a prejudice against the bark, contrayerva, and serpentary, were the principal remedies. Others administered cinchona, at first by itself, afterwards with wine, but both the compound, and its first ingredient, fell into disuse, though the employment of bark has again been revived, of late years, under the more effectual, and convenient, form, of sulphate of quinine. Camphor has been a favourite of

"* I cannot convey a better idea of this gratitude, than by repeating what often passed, when I questioned them, particularly on the day after admission, how they rested the preceding night; the answer has, innumerable times, been, 'Never better, sir; it is the only night I have had any sleep since I took it,'—the fever."

many. Cascarilla, angustura, and canella alba, have been occasionally prescribed, under the impression, that from their union of bitter and aroma, they might be of the same efficacy with the old serpentary, and contrayerva. I have repeatedly tried them all, and have found them of sufficient utility. Indeed, I have no doubt that any one of them will often conduct the disease to a prosperous termination. For many years back, however, I have laid them all aside, and trusted entirely, or almost entirely, to the use of wine. This remedy, in fact, possesses exactly the two powers necessary for correcting the pulse in typhus; restoring its strength in the mean time; and, as a prophylactic, preventing it from sinking afterwards. It, besides, possesses conveniences and advantages that other febrifuges want. Thus, it hardly ever disagrees with the stomach, a circumstance of great moment in a disease like fever, where the organ is so apt to be ticklish, or irritable. But its grand qualification consists in this, that you can derive from it every degree of stimulation you please, from the highest to the lowest, so as to command every grade of excitement you may deem requisite for your patient, and that, merely, by adjusting the doses, and abridging, or prolonging, the intervals of its exhibition. We have thus put into our hands not only a most powerful, but at the same time a most manageable instrument against fever. As to the mode of administration, I shall merely mention the plan I have long followed myself, and which, by experience, I have found the most advantageous. Unless great debility occur early in the disease I rarely prescribe it till the eighth, ninth, or tenth day, and then order it only in small quantities, and at considerable intervals, as half a glass or one ounce, every three hours; and I may here remark, once for all, that it must be managed in such a manner, that the succeeding may always support the influence, or impression, of the preceding dose. Should the artery assume a firmer beat, though still feebler than that of health, I make no augmentation, but proceed with the same allowance. If the pulse, on the contrary, continue too feeble, I increase the quantity, or shorten the interval of exhibition, ordering a glass every two hours, or half a glass every hour, or a whole glass every hour. For the most part, however, I rarely exceed twelve glasses, or an ordinary bottle, during the day and night. I must here warn you against a circumstance that might otherwise mislead you, it is this: it not unfrequently happens that when wine is taken in these large doses, though it invigorates the pulse to a certain extent, yet it does not raise it enough, and you might be apt to suppose that the medicine is given in vain. This, however, is not the case. It is keeping the disease at bay, and you have only to persevere, when, in a day or two, you will reap every benefit you desire. Occasionally, however, wine, even in considerable quantities, affords too weak a stimulant, and its power must be assisted by the addition of alcohol, under a stronger, or purer, form, as of our ordinary spirits, rum, whisky, or brandy. Half a glass of either may be added to each or every second dose of port, sherry, or madeira. If still the pulse flag, join sulphuric ether, as ten or fifteen drops, or more, to each exhibition of the other remedies; or interpose this powerful diffusible during the intervals. Should wine produce acidity, or otherwise disagree, spirits must be substituted in its room ; and it is needless to add, that every expedient must be tried to render whatever liquor you use agreeable to the stomach and palate. If possible too, or unless nausea forbid, some light aliment should be joined to your remedies, as beef tea, calf-foot jelly, or panada, with the addition of aromatics." (P. 50.)

"Before I quit this subject, I must not forget to tell you, that there are certain symptoms in low fever, according to many practitioners, which forbid the use of wine, and render it either useless or hurtful. What are esteemed the principal among these are a hot skin and dry tongue. There will be found here much fallacious reasoning among authors. If it be merely asserted that a fever is more difficult of cure with these symptoms than without them, the fact may be readily admitted, but they do not necessarily contraindicate the employment of wine. If the surface be moist as well as hot, the remedy is perfectly admissible, as I can assert from frequent trials, and if it be hot without being moist, the obstacle may be easily removed by sponging. But, in fact, a hot skin is rarely an accompaniment of our low typhus. With respect to dry tongue, I would rather it were moist, yet I give wine every day of my life, notwithstanding the circumstance, should the pulse demand it, not only without evil or aggravation of the disease, but with the most decided benefit to the sick. Cases often occur, indeed, in which there is no choice. If you find your patient living supine, passing his stools and urine in bed, and the pulse all but extinguished at the wrist, you will not do your duty unless you endeavour to restore, and support, him, by the plentiful use of the most powerful diffusibles, be the state of the tongue, and the skin, what it may.

"Among the London practitioners, ammonia, and the camphor mixture, separate, or conjoined, are favourite medicines, and without doubt they are of considerable efficacy. The first-named article has this advantage, that it determines to the skin, an influence always salutary in fever, but its stimulation is less permanent than that of wine. The best forms, I think, are either the simple or alcoholic solution, or that joined to some of the volatile aromatic oils, both of course to be administered in small, but repeated doses. Though in the above remarks a very high estimate has been given you of the febrifuge virtues of wine, you must not therefore suppose that I regard it as an absolute panacea, or that every person must recover, provided it be used in sufficient quantity. I trust I need not warn you against so gross an absurdity. Though much, no doubt, may be done for typhus by early and judicious management, yet universal immunity from its ravages can never be expected. Like every other epidemic, it must always have more, or fewer victims. Such is the baneful potency of typhus poison over the vital organs, more especially, it is probable, over the universal nervous tissue, that in many instances, prescribe as you may, no remedial means will avert the fatal event. Hence an invidious charge against wine, by saying that many of those die who have used it the most liberally. The fact is without question true, but the inference does not follow from the fact. No argument indeed can be more futile, since the same accusation might be brought, with equal strength, against every remedy our art can boast, even those of the most tried and acknowledged efficacy. But is a medicine to be reckoned useless merely because it is not omnipotent? Are copious blood-lettings, for example, with other depletions, and the antiphlogistic regimen, to be stigmatized as of no avail in inflammation, because every sthenic disease does not immediately fly before them, or because many persons are known to die under their administration? The same reasoning precisely is applicable to wine in typhus. The extent to which we are obliged to carry the stimulant plan in the one instance, and the depleting system in the other, merely shows the violence, and danger, of the disorder against which we have to contend. The amount of the whole is what may be readily conceded, that the best directed efforts of our art are too often nugatory, and that diseases are not unfrequently stronger than our remedies." (P. 53.)

We have already expressed our determination not to enter the wild regions of speculation with which the controversial section of this volume is filled; neither do we feel inclined to offer an opinion upon the political strictures contained in the Appendix: therefore, having given as copious an analysis of the practical part of these lectures as our limits would allow, we shall conclude our notice by recommending the work to the perusal of our younger readers; for it is evidently the production of one who has advantageously employed the extensive opportunities he has had of studying and practising his profession.

